# Saline nasal irrigations for chronic rhinosinusitis: From everyday practice to evidence-based medicine. An update

**DOI:** 10.1177/2058738418802676

**Published:** 2018-10-23

**Authors:** Manuele Casale, Antonio Moffa, Michele Cassano, Francesco Carinci, Michele Antonio Lopez, Eleonora Maria Consiglia Trecca, Sara Torretta, Vittorio Rinaldi, Lorenzo Pignataro

**Affiliations:** 1 Department of Otolaryngology, Campus Bio-Medico University, Rome, Italy; 2 Department of Otolaryngology, University of Foggia, Foggia, Italy; 3 Department of Morphology, Surgery and Experimental Medicine, University of Ferrara, Ferrara, Italy; 4 University of the Republic of San Marino, San Marino, San Marino; 5 Department of Otolaryngology and Department of Clinical Sciences and Community Health, Fondazione IRCCS Ca’ Granda Ospedale Maggiore Policlinico, University of Milan, Milan, Italy

**Keywords:** chronic rhinosinusitis, hypertonic solution, isotonic solution, saline nasal irrigation

## Abstract

Saline nasal irrigations (SNIs) are often recommended as an additional non-pharmacological treatment for adults with chronic rhinosinusitis (CRS), for which it could even be considered a first-line treatment. However, there is a wide range of different SNI protocols. The aim of this article is to review the published literature regarding all of the potential therapeutic effects of SNIs in adult CRS patients who had not undergone sinus surgery and clarify the role of the various saline nasal solutions and protocols (particularly the volume, frequency and duration of treatment), and describe the nasal devices used. A search was made of the PubMed, Google Scholar and Ovid databases using the key words ‘saline nasal irrigation’ and ‘chronic rhinosinusitis’, or medical subject headings. The search identified 11 studies involving 663 patients. There was no consensus about but substantial agreement concerning the frequency and duration of treatment, the type of device, and the amount of solution to be used when managing CRS. A hypertonic solution with the addition of the natural minerals and oligo-elements found in seawater and some thermal waters may be associated with greater clinical benefit in terms of endoscopic scores and mucociliary clearance than isotonic solutions. Further studies are required to compare the different forms of SNI and define SNI protocols and nasal devices, while considering patient compliance.

## Introduction

Epidemiological studies have shown that chronic rhinosinusitis (CRS) affects 10%–15% of the people in the United States with peaks between 30 and 60 years of age. CRS is characterised by mucosal inflammation of the nose and paranasal sinuses and can be divided into two broad clinical categories: CRS with and without nasal polyposis.

The most widely used means of treating CRS are topical nasal sprays, oral steroids and antibiotics, and saline nasal irrigations (SNIs), which is often recommended for CRS patients in everyday clinical practice. SNIs are most frequently carried out using isotonic or hypertonic saline or seawater solutions, and typically improve nasal mucosa function as a result of direct mucosal cleansing; the removal of antigens, biofilms or inflammatory mediators (thus resolving inflammation) and improved mucociliary function.

However, SNI protocols vary widely in terms of the volume, frequency and duration of treatment, and nasal devices used. A 2016 Cochrane review concluded that a low-volume (5 mL) nebulised saline spray offered no benefit over intranasal steroids, but that daily, large-volume (150 mL) irrigations with a hypertonic saline solution were more beneficial than placebo, although the quality of the evidence was low. However, as this review gave no information concerning tonicity, volume, delivery, frequency or duration of use, and included only two very small, open-label and clinically heterogeneous studies with major limitations, it is difficult to draw any practical conclusions.^1^

The aims of this review were to verify the effectiveness of SNIs in CRS patients using the criteria of evidence-based medicine and clarify the roles of the various saline solutions; the volume, frequency and duration of treatment; and the types of nasal devices.

## Methods

### Search strategy and article selection process

A search was made of the PubMed, Google Scholar and Ovid databases in accordance with the PRISMA guidelines using the following key words or (in the case of PubMed) medical subject headings: ‘nasal washes’, ‘nasal irrigation’, ‘nasal douche’, ‘saline nasal irrigation’, ‘saline solution’, ‘sodium chloride solution’, ‘isotonic solution’, ‘hypertonic solution’, ‘thermal water solution’, ‘seawater solution’ and ‘chronic rhinosinusitis’.

The main eligibility criteria were English-language articles, randomised and controlled trials in humans and the effect size of SNI evaluated clinically in patients with CRS symptoms who had not undergone sinus surgery. There were no restrictions in terms of date of publication or study duration, but retrospective studies, literature reviews, technical notes, letters to editors and instructional courses were excluded, as were paediatric studies, studies that simultaneously considered episodes of acute and CRS, and studies of the use of SNI following endoscopic sinus surgery. Additional literature was found by reviewing the reference lists of the selected articles. The authors then independently assessed the full-text versions of each publication and excluded those whose content was judged not to be strictly related to the subject of this review.

## Results

### Analysis of the literature

**Figure 1.**
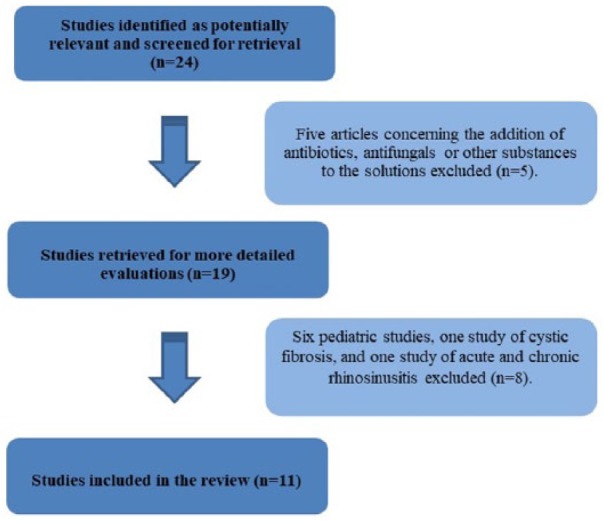
Flow chart of study selection process.

The search initially identified 24 potentially relevant studies but, after excluding five studies concerning the addition of corticosteroids, antibiotics, antifungals or polysaccharides to normal saline solution, six paediatric studies, one study of cystic fibrosis, and one study of acute and CRS, the final analysis was based on 11 studies involving a total of 663 patients (Figure 1): eight clinical trials comparing different saline solutions and three comparing the effects of nasal solutions delivered by means of different devices.

### Clinical studies of the therapeutic effects of different saline nasal solutions

#### Thermal water solution versus isotonic saline solution

Ottaviano et al.^2^ evaluated the effects of 30 days’ SNI on 70 smokers with non-allergic CRS: 35 treated with sulphurous–arsenical–ferruginous thermal water and 35 treated with an isotonic saline solution. During the follow-up, all of the participants underwent n-butanol olfactory threshold tests, nasal cytology, active anterior rhinomanometry and nasal endoscopy.

The group treated with thermal water showed a statistical trend towards lower nasal resistances and significantly greater improvements in the number of ciliated cells and nasal endoscopy results after 1 month, but the olfactory threshold was significantly higher in the group treated with the isotonic saline solution.

#### Sulphurous, salty, bromic, iodic thermal water versus isotonic saline solution

Ottaviano et al.^3^ compared the effects of 30 days’ SNI on 80 CRS patients: 40 treated with sulphurous, salty, bromic, iodic (SSBI) thermal water and 40 treated with an isotonic saline solution. Upon enrolment and at the end of the treatment, the patients provided microbiological nasal swabs and underwent anterior active rhinomanometry and nasal endoscopy.

Nasal endoscopy showed a significant clinical improvement in both groups at the end of the treatment, and there were no signs of bacteria, but only the SSBI water irrigations significantly reduced total nasal resistance.

#### Hypertonic versus isotonic seawater solution

Culig et al.^4^ compared the efficiency of 15 days’ treatment with a 0.9% isotonic or a 2.12% hypertonic seawater solutions in 60 CRS patients (30 in each group). Both solutions contained trace amounts of natural minerals and oligo-elements, but the hypertonic solution was enriched with monohydrated manganese and pentahydrated copper salts. The patients were asked to write notes concerning their sinonasal symptoms and to self-complete a quality of life questionnaire covering the quality of sleep, and their daily activities and emotions. The hypertonic solution was significantly better, especially in terms of nasal congestion, rhinorrhea, cough, headache and waking up during the night.

#### Buffered isotonic solution versus buffered hypertonic solution

Hauptman and Ryan^5^ compared the effects of single administrations of 1 mL of buffered isotonic solution (0.9%) or buffered hypertonic solution (3%) delivered by means of a standard nasal spray on 80 CRS patients (40 in each group). Before and after the treatment, the patients were interviewed about their main nasal symptoms and underwent anterior rhinomanometry and saccharine clearance tests.

Both solutions significantly improved nasal symptoms and mucociliary clearance, which was greater after the administration of the hypertonic solution. However, the hypertonic solution had no effect on nasal patency, which was increased by the isotonic solution.

#### Dead Sea salt solution versus hypertonic saline solution

Friedman et al.^6^ investigated the effects of 60 days’ treatment with a 1.8% hypertonic Dead Sea salt solution (DSS, 22 patients) or an 1.8% hypertonic saline solution (20 patients). Before enrolment and 30 days after treatment, all of the patients underwent anterior rhinoscopy and nasal endoscopy and completed a 16-point rhinitis symptom questionnaire and the standardised Rhinoconjunctivitis Quality of Life Questionnaire (RQLQ(S)). SNI with DSS significantly improved rhinitis symptoms and RQLQ(S) scores in comparison with the hypertonic saline solutions.

#### Hypertonic SNI using 2% buffered saline in a SinuCleanse nasal cup

Rabago et al.^7^ investigated the efficacy of hypertonic saline nasal irrigation (HSNI) in 54 CRS patients: 40 treated with HSNI using 2% buffered saline in a SinuCleanse nasal cup and 14 not undergoing nasal washes. During the follow-up, clinicians used the Rhinosinusitis Disability Index (RSDI), a sinus-symptom severity assessment (SIA) and the Sino-Nasal Outcomes Test 20 (SNOT-20) and evaluated the frequency and pattern of HSNI, its side effects and the patients’ satisfaction.

In the HSNI group, RSDI scores continued to improve, while SIA and SNOT-20 scores remained stable. HSNI was used for a mean of 2.4 irrigations per week; 33% of the patients used HSNI regularly, and 55% when symptomatic. There were only minor side effects, and patient satisfaction was high.

#### Daily hypertonic SNI improves sinus-related quality of life and decreases medication use

Rabago et al.^8^ evaluated the efficacy of daily hypertonic SNI for 6 months in 52 CRS patients and compared the results with those observed 24 CRS patients not undergoing SNI. Clinicians administered the short form of the Medical Outcomes Survey (SF-12), the RSDI and the single-item sinus-symptom severity assessment (SIA) and assessed compliance daily, and symptoms and the use of medication every 2 weeks.

The RSDI and SIA scores in the SNI group improved in comparison with the control group, and the subjects reported fewer 2-week periods with sinus-related symptoms and used antibiotics and nasal sprays less frequently.

#### Ems salt solution versus isotonic saline solution

Bachmann et al.^9^ compared the effectiveness of 7 days’ SNI using Ems salt thermal water (1.1%, and rich in Rb+, Cs+, Ba2+, Mn2+, and Sr2+) in 20 CRS patients and isotonic saline solution in 20 CRS patients. All of the subjects underwent nasal endoscopy, plain radiography of the paranasal sinuses, olfactometry, anterior rhinomanometry and a saccharin-clearance test on days 1 and 7, and the patients kept a diary in which to record general discomfort, nasal airway obstruction, and the use of additional nasal spray.

Olfactometry, saccharine clearance and rhinomanometry values were slightly but non-significantly better in the Ems salt solution groups than in the controls. General discomfort improved in both groups, but the control group more frequently required the additional use of decongestive nasal sprays.

### Clinical studies of the therapeutic effects of different means of administration

#### Large-volume isotonic SNI at low positive pressure versus isotonic spray

Pynnonen et al.^10^ compared the effects of 8 weeks of treatment with a large volume of isotonic SNI solution delivered at low positive pressure (61 CRS patients) with those of isotonic sprays (60 patients). Clinicians evaluated symptom severity using the SNOT-20, as well as symptom frequency and changes in the use of medications.

The irrigation group had lower SNOT-20 scores after 2, 4 and 8 weeks and showed a significant reduction in symptom frequency in comparison with the control group. There was no significant between-group difference in the use of sinus medication.

#### SNI using a bulb syringe versus SNI using a nasal irrigation pot

Heatley et al.^11^ evaluated the therapeutic effects of SNI on CRS adult patients divided into three groups of 50 patients each: groups 1 and 2 underwent daily hypertonic SNIs using a bulb syringe for 2 weeks followed by further 2 weeks using a nasal irrigation pot, or vice versa; group 3 did not undergo nasal irrigation. All of the patients completed pretreatment Medical Outcomes Study Short Form, pretreatment and posttreatment Rhinosinusitis Outcomes Measure, and a record was made of their daily medication use, subjective judgements of treatment efficacy, and preferred irrigation method.

There was a significant and equivalent improvement in RSOM31 score after 2 weeks of treatment in groups 1 and 2, and a total of 35% of the subjects reported a decrease in their use of decongestants, antihistamines, pain relievers, and nasal sprays, with no measurable difference between the three groups.

#### Alkaline nasal douche versus seawater spray

Taccariello et al.^12^ compared 8 weeks of treatment using an alkaline nasal douche with a 1:1 mixture of sodium chloride and sodium bicarbonate (19 patients) or a sterile seawater spray (21 patients); a control group of 22 patients did not undergo nasal washes. At the beginning and end of the treatment period, the patients underwent rigid endoscopy and acoustic rhinometry, and nasal mucociliary clearance and ciliary beat frequency tests; they also completed a symptoms diary card and a quality of life questionnaire.

There were significant differences between the two treatment groups insofar as the alkaline nasal douche improved endoscopic findings but not quality of life scores, whereas the opposite was true for the spray. There were no significant between-group differences in the acoustic rhinometry findings, diary card scores, nasal mucociliary clearance or ciliary beat frequency tests.

## Discussion

**Figure 2.**
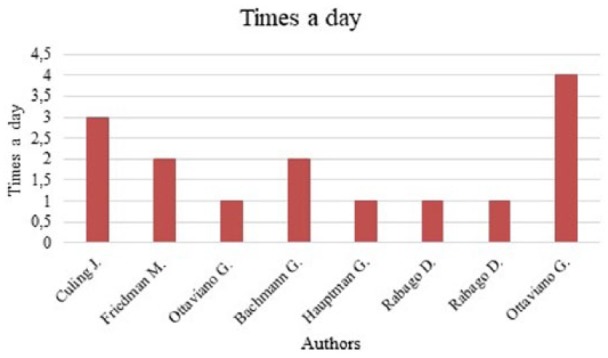
Frequency of treatment.

**Figure 3.**
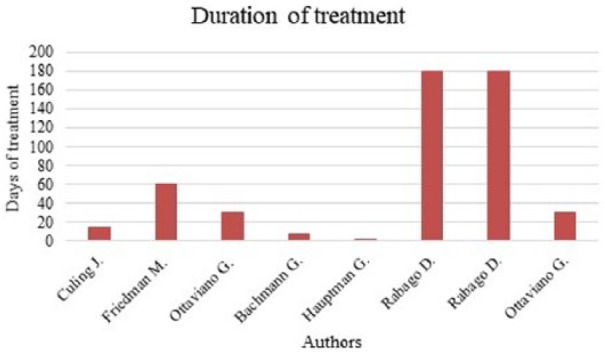
Duration of treatment.

Founded on everyday practice and common sense, SNI plays an essential role in the CRS medical treatment for the large majority of practitioners. In this review, six studies evaluated the use of SNI once or twice a day, and two its use more than three times a day (Figure 2). Treatment duration varied from a single administration^5^ to 6 months^7,8^ (Figure 3). Only four studies^2,3,5,9^ specified the dose of the daily administrations. Two clinical trials used sprays, and six simply specified ‘irrigation’. Hauptman et al.^5^ and Bachmann et al.^9^ found that hypertonic solutions improved mucociliary clearance better than isotonic solutions, and Ottaviano et al.^2^ and Bachmann et al.^9^ observed that they also led to a greater improvement in nasal resistance as assessed by means of active anterior rhinomanometry. Hypertonic seawater solutions also provided significantly better symptom relief in terms of nasal congestion, rhinorrhea, cough and headache than isotonic solutions.^4,5^

Despite the heterogeneity of the studies, there was a trend towards the conclusion that the once- or twice-daily administration of a hypertonic solution with the addition of the natural minerals and oligo-elements found in seawater and some thermal waters may be associated with a greater clinical benefit, better endoscopic scores and improved nasal resistance than isotonic solutions.^2-6^

SNI is inexpensive, can be performed at home and is a good treatment option for many patients. It is rarely accompanied by adverse effects, although the use of hypertonic solutions can lead to the irritation of nasal mucosa and a greater sensation of burning.^5^ The findings of the studies of different delivery methods and devices were somewhat conflicting.

In conclusion, the few studies of the use of SNI in CRS patients are characterised by a small patient populations, short observation periods and different clinical and diagnostic parameters evaluated. More studies are required to identify the best means of administration (spray, syringe, nasal pot, spray-sol, etc.) and the best treatment schedule. The close connection between particle diameter and high concentrations of nebulised particles in the upper aero-digestive tract suggests the need to choose nebulisers carefully in order to obtain better therapeutic results. Tailored SNI for CRS patients should not only consider the solution used, the most suitable device, and the most appropriate treatment schedule, but also patient compliance, which is crucial in the case of daily treatment for a chronic inflammatory disease such as CRS.
